# Structure of the Inhibited Smooth Muscle Myosin and Its Implications on the Regulation of Insect Striated Muscle Myosin

**DOI:** 10.3390/life15030379

**Published:** 2025-02-27

**Authors:** Shaopeng Sun, Yi-Ning Lu, Xiang-dong Li

**Affiliations:** 1 Group of Cell Motility and Muscle Contraction, State Key Laboratory of Integrated Management of Insect Pests and Rodents, Institute of Zoology, Chinese Academy of Sciences, Beijing 100101, China; sunshaopeng@ioz.ac.cn (S.S.); luyining23@ioz.ac.cn (Y.-N.L.); 2 University of Chinese Academy of Sciences, Beijing 100049, China

**Keywords:** alternative splicing, insect, myosin, regulation, striated muscle myosin

## Abstract

Class II myosin (myosin-2) is an actin-based motor protein found in nearly all eukaryotes. One critical question is how the motor function of myosin-2 is regulated. Vertebrate myosin-2 comprises non-muscle myosin, smooth muscle myosin and striated muscle myosin. Recent studies have shown that smooth muscle myosin, in its inhibited state, adopts a folded conformation in which the two heads interact with each other asymmetrically, and the tail is folded into three segments that wrap around the two heads. It has been proposed that the asymmetric head-to-head interaction is a conserved, fundamental structure essential for the regulation of all types of myosin-2. Nearly all insects have only a single striated muscle myosin heavy chain (MHC) gene, which produces all MHC isoforms through alternative splicing of mutually exclusive exons. Most of the alternative exon-encoded regions in insect MHC are located in the motor domain and are critical for generating isoform-specific contraction velocity and force production. However, it remains unclear whether these alternative exon-encoded regions participate in the regulation of insect striated muscle myosin. Here, we review the recently resolved structure of the inhibited state of smooth muscle myosin and discuss its implications on the regulation of insect striated muscle myosin. We propose that the alternative exon-encoded regions in insect MHC not only affect motor properties but also contribute to stabilizing the folded conformation and play a crucial role in regulating insect striated muscle myosin.

## 1. Introduction

Myosins are a superfamily of motor proteins that are capable of converting chemical energy from ATP hydrolysis into mechanical energy, producing movement along actin filaments (F-actin). Class II myosin (myosin-2) serves as a molecular motor that works in conjunction with F-actin using energy derived from the hydrolysis of ATP to power diverse essential biological processes such as muscle contraction, cell division, cell adhesion, and cell migration [1,2]. Myosin-2 can be roughly divided into non-muscle myosin, smooth muscle myosin (SmM) and striated muscle myosin [3,4]. Myosin-2 is a hexamer consisting of two heavy chains, two essential light chains (ELCs) and two regulatory light chains (RLCs). Each heavy chain contains a globular head that possesses actin-binding and ATPase activities, a lever arm stabilized by one ELC and one RLC and a C-terminal α-helical tail that dimerizes the two heavy chains to form a coiled-coil tail of the hexamer [5].

SmM, as well as non-muscle myosin, is activated by phosphorylation of the RLC. When the RLC is dephosphorylated, SmM is in the inhibited state, with very low ATPase activity. Electron microscopy shows that the inhibited SmM forms the folded conformation, in which the two heads fold back to the proximal tail portion and are wrapped by the distal tail portion [6]. Upon RLC phosphorylation, SmM forms an extended conformation and exhibits high ATPase activity.

On the other hand, the activity of striated muscle myosins, including skeletal muscle myosin and cardiac muscle myosin, regardless of RLC phosphorylation, is controlled by troponin/tropomyosin in a calcium-dependent manner. Nevertheless, RLC phosphorylation does play a modulatory role in cardiac muscle myosin [7]. Three-dimensional reconstruction of the electron microscopy images reveals the folded molecular feature in the relaxed (inhibited) state of *Tarantula* leg muscle and cardiac muscle myosin [8,9,10,11,12]. In cardiac muscle myosin, the tails intertwine to form filaments, exposing the conserved interacting heads motif (IHM) on their surface. The IHM structure in cardiac muscle myosin, like that in SmM, stabilizes the relaxed state, and interactions between different IHMs may further reinforce this state. Cryo-EM analysis shows that while RLC phosphorylation regulates the SmM IHM conformation, the IHM in cardiac myosin is not fully regulated in the same manner, warranting further investigation into its specific regulation. Despite some regulatory differences between smooth and cardiac muscle, the shutdown state and folded conformation are conserved across all myosin-2 paralogs [13,14,15]. It has been proposed that the folded conformation is a fundamental structure critical to the function and regulation of both striated and smooth muscle myosins across a wide range of species [4,9,12,16].

Unlike vertebrates, which possess multiple striated muscle myosin heavy chain (MHC) genes, almost all insects have only a single *Mhc* gene that produces all MHC isoforms as a result of alternative splicing of mutually exclusive exons [17,18,19]. For example, the *Drosophila Mhc* gene is capable of encoding 480 different MHC isoforms by using various combinations of alternative exons. Multiple *Drosophila Mhc* transcripts have been identified in different muscles, including transverse (external) muscle, body wall muscle, intermediate muscle and visceral muscle [20]. These different types of myosin are expected to have distinct properties to support diverse physiological functions of the muscle. Genetic and biochemical studies of transgenic *Drosophila* indicate that the alternative exon-encoded regions in the motor domain are critical for generating isoform-specific contraction velocity and force production [18,21,22]. However, how they tune the regulation of the myosin molecule and how they further lead to different physiological properties of different insect MHCs remain unknown. In addition, the interaction between the two dephosphorylated RLCs plays a critical role in the formation and stabilization of the inhibited conformation of SmM. Although the RLC–RLC interaction interface in striated muscle myosin differs somewhat from that in SmM, it might also contribute to the formation and stabilization of the IHM of striated muscle myosin. However, due to the lack of high-resolution structures of striated muscle myosin, how RLC phosphorylation regulates its activity requires further exploration.

In this paper, we reviewed the recent advances in the structure of the inhibited state of smooth muscle myosin and discussed its implications on the regulation of insect striated muscle myosin, based on the high structural resolution of its folded conformation and its strong conservation across all myosin-2 paralogs. We propose that the alternative exon-encoded regions in the motor domain of insect striated muscle not only affect the motor properties per se but also regulate the conformation and active state of the myosin, with BH exon 7 and FH exon 9 as the most critical.

## 2. Structure of the Inhibited Smooth Muscle Myosin

**Figure 1:**
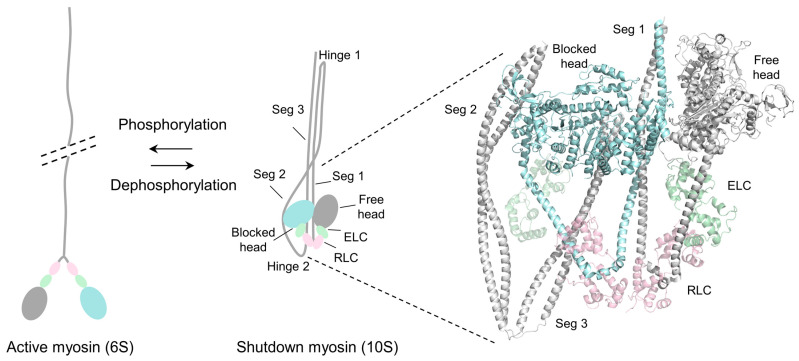
Schematic drawing of the conformational transition of SmM and the 3D structure of the IHM of shutdown SmM. The left panel shows the conformational transition between the 6S active state and the 10S shutdown state of smooth muscle myosin. The right panel is the entire folded molecule showing IHM and the folded tail (PDB ID: 7MF3, 3.4 Å). Color code: light blue, blocked head; gray, free head; light gray, tail; light green, ELC; light pink, RLC.

The motor activity and the conformation of SmM are regulated by the phosphorylation of serine 19 (S19) on the RLC [23]. The unphosphorylated form of SmM is inactive (or inhibited), i.e., it has low actin-activated ATPase activity and is incapable of moving actin filaments in vitro, whereas the phosphorylated form is active in both respects (Figure 1).

SmM exists in two conformations: the 10S folded conformation and the 6S extended conformation [24]. The inhibited SmM forms the 10S folded conformation, in which the two heads interact with each other asymmetrically to form an interacting heads motif (IHM), while the tail is folded into three segments, wrapping around the two heads [6]. In the IHM, the actin-binding site of the “blocked” head (BH) is blocked by the converter domain of the “free” head (FH) [25,26]. Upon RLC phosphorylation, SmM is activated and adopts the 6S extended conformation, in which the two heads interact with F-actin and hydrolyze ATP and the coiled-coil tail self-associates to polymerize into thick filaments. The folded conformation prevents filament formation and inactivates the motors, serving as a key energy-conserving form in cells [27,28,29,30]. Detailed structural information is essential to understand the activation and inhibition of myosin-2.

Three recent studies using single-particle cryo-electron microscopy have determined the high-resolution structure of the folded conformation of the inhibited SmM, providing a framework for understanding the regulation of myosin-2 [13,31,32]. The folded conformation of SmM is stabilized by multiple intramolecular interactions that take place in mainly four interfaces: the head–head interface, the BH–tail interface, the FH–tail interface, and the RLC–RLC interface (Figure 1).

The BH–FH interface is the primary contact region that stabilizes the IHM. A network of electrostatic interactions and hydrogen bonds forms between BH loop 4, helix M, FH relay and converter. The BH CM loop is also blocked by the HO linker and helix HE of FH. In addition, the actin-binding surface of BH, consisting of loop 2, loop 3, loop 4 and the helix–loop–helix region, is blocked by extensive interactions with FH. Furthermore, the electrostatic interactions between loop 2 of BH and segment 1 also participate in the stabilization of the shutdown state. Moreover, the BH ATP-lip contacts with the FH converter by a key hydrophobic interaction.

Mainly three interactions are formed in the BH–tail interface, in which segment 2 of the tail interacts with the N-SH3, the relay and the converter domain of BH. It directly reveals that the inhibited ATPase activity of BH is due to the physical blockage of the converter movement, which is required for Pi release. The FH–tail interface includes two interactions that partially decrease the actin-binding affinity of FH: the CM loop and loop 2 of FH, which are part of the actin-binding interface, are blocked by extensive ionic interactions with segment 1.

The RLC–RLC interface plays a critical role in the formation and stabilization of the folded conformation and is a key contributor to the regulation of myosin-2. The N-terminals of the RLCs interact with each other by a network of interactions. In addition, helix E of BH RLC lies near segment 3 and forms ionic interactions, while helix A in the N-lobe of the FH RLC contacts segment 1, potentially stabilizing the shutdown state. The interactions between ELCs and RLCs, as well as between BH ELC and segment 2 also contribute to the stabilization of the inhibited SmM.

The activation of SmM is regulated by RLC phosphorylation at S19, catalyzed by myosin light chain kinase (MLCK). RLC phosphorylation allows the release of the shutdown state, promotes filament formation and finally activates myosin motor activity. Phosphorylation of both of the two RLCs is required for full activation of SmM [33]. In the IHM, the positions of the N-terminal extensions of the RLCs of BH and FH are different, consistent with the observation that the two RLCs in SmM are phosphorylated sequentially [34]. However, there is controversy over whether the RLC of the FH or the BH is phosphorylated first [13,31]. Scarff et al. [13] proposed that the FH RLC may be phosphorylated first. According to their IHM model, while both RLC N-terminal extensions are surface exposed, the BH RLC is less accessible due to juxtaposing to the folded tail. On the other hand, Heissler et al. [31] suggested that phosphorylation first occurs on the RLC of BH, because the RLC S19 of BH is exposed, while that in the FH is buried in the RLC–RLC interface. Further research is necessary to clarify this issue.

The high-resolution structure of the folded conformation of the inhibited SmM also provides a framework for understanding the regulation of other myosin-2s. The structural similarities between smooth muscle myosin and muscle myosin-2 suggest that the IHM identified in the inhibited state of smooth muscle myosin may be broadly applicable across various myosin-2 isoforms. Smooth muscle myosin, when in its inhibited folded conformation, exhibits specific intramolecular interactions and structural motifs that stabilize its inactive state. The asymmetric head-to-head interaction and the head–tail interaction are likely conserved in other myosin-2 isoforms due to their shared evolutionary origins and functional requirements.

## 3. Regulatory Role of the Alternative Regions in Insect Striated Muscle Myosin

The IHM was proposed to be evolved about 800 million years ago in the ancestor of Metazoa [35]. Cryo-EM studies have revealed that the myosin IHM (including an asymmetric head-to-head interaction and interaction of S2 with the BH in filaments) is present in the relaxed thick filaments isolated from *Tarantula* leg muscle [9,12,36] and the scorpion striated muscle [37]. However, an exception is observed in the *Lethocerus* indirect flight muscle myosin, which adopts a unique inhibited conformation within the relaxed thick filament, characterized by a perpendicular IHM that does not fold back to contact its own S2 [38]. Notably, the *Lethocerus* indirect flight muscle is a highly specialized muscle and may not be representative of the non-flight muscle myosin. It is therefore likely that the IHM is conserved in the muscle myosin of arthropods, including insects, suggesting a broader preservation of this structural motif across species.

**Figure 2:**
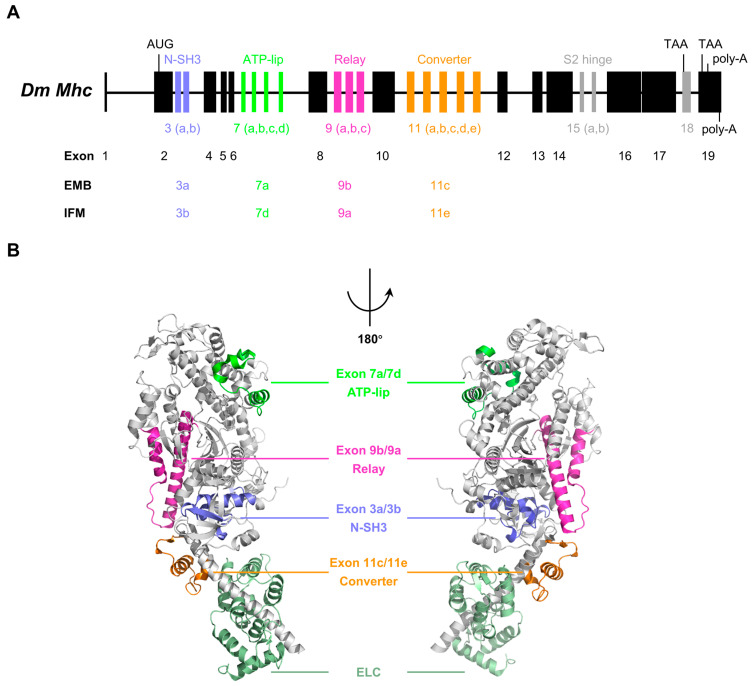
Diagram of the *Drosophila Mhc* gene and the structure of *Drosophila* myosin-2. (**A**) Diagram of the *Drosophila Mhc* gene, showing the exon–intron structure. Constitutive exons are colored in black, and alternative exons are colored. The exon usage of typical isoforms, such as embryonic muscle myosin (3a/7a/9b/11c) and indirect flight muscle myosin (3b/7d/9a/11e), is displayed. (**B**) Crystal structure of the *Drosophila* myosin-2 motor domain (PDB ID: 5W1A, 2.23 Å), exhibiting the alternative exon usage in embryonic muscle myosin (3a/7a/9b/11c) and indirect flight muscle myosin (3b/7d/9a/11e). The alternative regions in the structure are color-coded to correspond to the exons in the *Drosophila Mhc* gene. Color code: gray, motor head; light green, ELC; blue, exon 3 (N-SH3); green, exon 7 (ATP-lip); magenta, exon 9 (relay); orange, exon 11 (converter).

Unlike vertebrates having multiple striated muscle myosin heavy chain genes, almost all insects have only a single *Mhc* gene that produces all MHC isoforms as a result of alternative splicing of mutually exclusive exons [17,18,19]. For example, the *Drosophila Mhc* gene consists of 13 constitutive exons, 5 clusters of alternatively spliced exons (Exon 3, 7, 9, 11 and 15) and 1 differentially included penultimate exon (Exon 18) (Figure 2A). In theory, the *Drosophila Mhc* gene is capable of encoding 480 different types of MHC protein by using different combinations of the alternative exons. Among those alternative exons, four clusters of alternative exons (Exon 3, 7, 9 and 11) are located in the motor domain of the gene (Figure 2A,B), potentially encoding 120 different variants of the motor domain. Exon 15 and Exon 18 are located in the tail domain, respectively (Figure 2A). A similar exon–intron pattern was found in the *Mhc* gene of other insects, except that some of them contain additional clusters of alternatively spliced exon [17]. Selection of alternatively spliced exon variants is developmentally specific and tissue specific [20,39].

The crystal structure of *Drosophila* myosin-2 motor domain (Figure 2B) reveals the locations of the four alternative regions in the motor domain of *Drosophila* MHC, including part of the N-SH3 subdomain encoded by exon 3, a region near the ATP-binding pocket (ATP-lip) encoded by exon 7, the relay encoded by exon 9, and part of the converter encoded by exon 11 [40,41]. The exon usage of typical isoforms, such as embryonic muscle myosin (3a/7a/9b/11c) and indirect flight muscle myosin (3b/7d/9a/11e), has been characterized (Figure 2A,B). Genetic and biochemical studies of transgenic *Drosophila* demonstrate that these alternative exon-encoded regions in the motor domain are critical for generating isoform-specific contraction velocity and force production [18,21,22].

**Figure 3:**
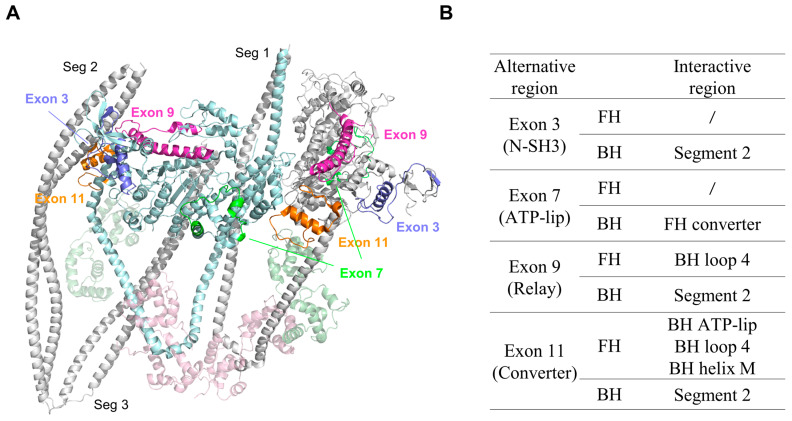
Localization and interactions of the four alternative regions in the motor domain of *Drosophila* MHC. (**A**) Structure of the IHM of shutdown myosin-2, highlighting the location and the interactions of the four alternative regions. Color code: light blue, blocked head; gray, free head; light gray, the tail; light green, ELCs; light pink, RLCs; blue, exon 3 (N-SH3); green, exon 7 (ATP-lip); magenta, exon 9 (relay); orange, exon 11 (converter). (**B**) Summary of interactions involving the alternative regions in the IHM.

As discussed above, the folded conformation of SmM is stabilized by the asymmetric head-to-head interaction and the head–tail interaction. We mapped the four alternative regions in the motor domain of *Drosophila* MHC onto the SmM IHM structure (Figure 3A). In the 3D structure of the inhibited SmM, these four regions are located at the binding interfaces between the heads and between the head and the tail. It is possible that these four alternative regions in the *Drosophila* MHC motor domain might participate in interactions that stabilize the folded conformation, thus regulating the motor function of insect striated muscle myosin.

**Exon 3-encoded region**

**Figure 4:**
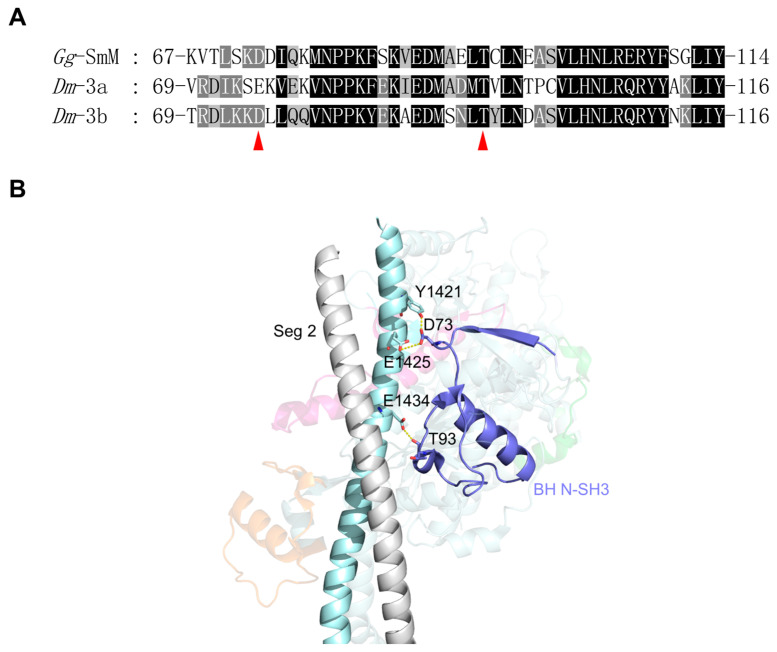
Amino acid sequence and interaction analyses of exon 3 encoding region. (**A**) Amino acid sequence alignment of chicken smooth muscle myosin and *Drosophila* MHC in the regions encoded by the alternative exon 3 of the *Drosophila Mhc* gene. The red arrows indicate the amino acids in BH exon 3 that are involved in stabilizing the IHM. (**B**) Interactions between the BH N-SH3 and segment 2. The color coding is consistent with Figure 3.

The *Drosophila Mhc* gene contains two exon 3 variants (3a and 3b), which encode amino acid residues 69 through 116 of the MHC. Exon 3a is mostly expressed in EMB, including internal wall muscles, visceral muscles, and embryonic cardioblasts, while exon 3b is majorly expressed in IFM [20]. Transgenic *Drosophila* shows that exchanging the embryonic exon 3a region into the flight muscle MHC isoform significantly reduced ATPase rates, slightly impaired flight ability and had little effect on actin gliding velocity [21]. The exon 3-encoded region is located in a portion of the N-SH3 subdomain, beginning within a β-sheet and winding its way through a long loop and an α-helix before ending within the first β-sheet of the transducer (Figure 4). This region is in proximity to the SH-1 helix. Thus, the variable amino acid residues encoded by exon 3 variants could affect the movement of the SH-1 helix during ATP hydrolysis and the twisting of the β-sheet of the transducer.

In the IHM, BH N-SH3 and FH N-SH3 have different effects on stabilization of the folded conformation. While FH N-SH3 does not participate in the folded conformation, BH N-SH3 forms several electrostatic interactions with segment 2 of the tail (Figure 4B). Segment 2 passes through a groove on the edge of the BH formed by the SH3 domain, the SH-1 helix and the converter domain. Electrostatic interactions are formed between D73 of N-SH3 and Y1421/E1425 of segment 2 and between T93 of N-SH3 and E1434 of segment 2 (Figure 4B). The residues involved in these interactions in N-SH3 are conserved between exon 3a and 3b, except for D73, which is substituted by Glu in exon 3a. This change may affect the interaction between BH N-SH3 and segment 2, thus altering the stability of the folded conformation. Because exchanging exon 3a region into the flight muscle MHC isoform significantly decreased ATPase rates [21], we expect that the exon 3a region interacts more strongly with segment 2 than the exon 3b region.

**Exon 7-encoded region**

**Figure 5:**
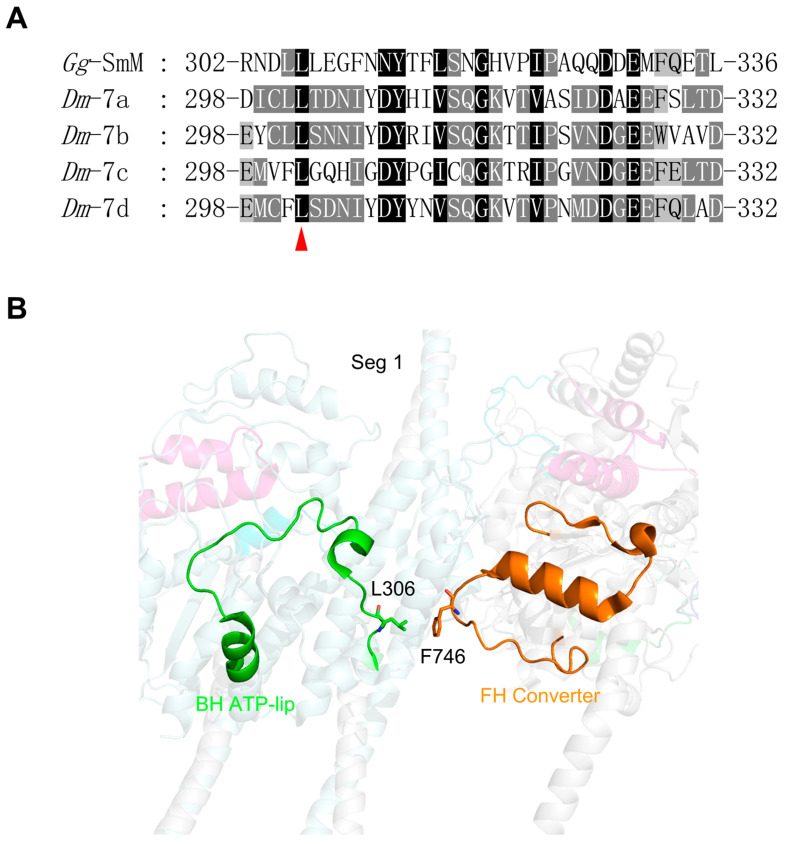
Amino acid sequence and interaction analyses of exon 7 encoding region. (**A**) Amino acid sequence alignment of chicken smooth muscle myosin and *Drosophila* MHC in the regions encoded by alternative exon 7 of the *Drosophila Mhc* gene. The red arrow indicates the amino acid in BH exon 7 involved in stabilizing the IHM. (**B**) Interaction between the BH ATP-lip and the FH converter. The color coding is consistent with Figure 3.

The *Drosophila Mhc* gene contains four exon 7 variants (7a, 7b, 7c and 7d), which encode amino acid residues 298 through 332 of the MHC. The exon 7-encoded region forms a helix-loop-helix, which resembles a circular structure (Figure 5B). It is located on the surface of the U50 subdomain directly above the nucleotide-binding pocket, forming one lip of the ATP-binding pocket. Thus, we name the exon 7-encoded region the ATP-lip. Exon 7a is usually used in EMB, while 7d is in IFM [20]. There are twelve amino acids within the exon 7a encoded region that differ from the exon 7d encoded region. The exon 7 region is adjacent to the nucleotide pocket and is expected to mediate nucleotide access to the active site [41]. Nevertheless, both the substitution of 7d into the EMB and the substitution of 7a into the IFM only slightly increase the maximal ATPase activity but have no effect on actin filament velocity [42,43].

In the IHM, the FH exon 7 region does not participate in the formation of the folded conformation, while the BH exon 7 region is located in the BH–FH interface, where L306 of BH forms a hydrophobic interaction with F746 of FH (Figure 5B). L306 is absolutely conserved among all exon 7 variants of *Drosophila* MHC (Figure 5A), highlighting the essential role of the bulky hydrophobic residue at this position. Moreover, the hydrophobic residue F746 is not conserved in the alternative exon 11 regions of *Drosophila* MHC, suggesting a regulatory role of exon 11 (see below).

**Exon 9-encoded region**

**Figure 6:**
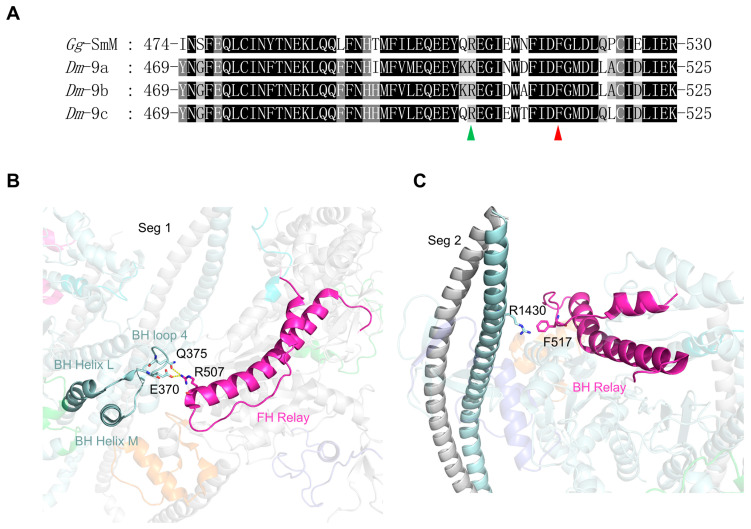
Amino acid sequence and interaction analyses of exon 9 encoding region. (**A**) Amino acid sequence alignment of chicken smooth muscle myosin and *Drosophila* MHC in the regions encoded by alternative exon 9 of the *Drosophila Mhc* gene. The red arrow indicates the amino acid in BH exon 9 involved in stabilizing the IHM, and the green arrow indicates the amino acid in FH exon 9 involved in stabilizing the IHM. (**B**) Interactions between the FH relay and BH loop 4. (**C**) Interaction between the BH relay and segment 2. The color coding is consistent with Figure 3.

The *Drosophila Mhc* gene contains three exon 9 variants (9a, 9b and 9c), encoding residues 469 through 525 of *Drosophila* MHC. Exon 9a and 9b are expressed in IFM and EMB, respectively. Studies of transgenic *Drosophila* show that exchanging exon 9b of EMB into the flight muscle isoform results in no significant difference in motility or ATPase activity compared to IFM myosin [22]. However, the EMB isoform with the IFM exon 9b decreased actin-activated ATPase activity. The exon 9 region forms a helix-loop-helix structure, which corresponds to the entire relay region of MHC (Figure 6B,C). By connecting the L50 and converter subdomain, the relay transmits the conformational changes in the active site to the converter-lever arm system [44]. The amino acid sequences in exon 9 variants are highly conserved, with a few exceptions at specific positions (Figure 6A).

The alternative exon 9 variants affect the basic mechanical properties of myosin, including power output, kinetics of power generation, tension generation and muscle stiffness [22,45,46]. Located at the distal end of the relay, three residues (I508, N509 and D511) interact with R759 of the converter [46]. Among them, the interaction mediated by the residues 509 and 759 was shown to be essential for the motor function of myosin, as well as for the normal ultrastructural and mechanical properties of *Drosophila* muscle [47,48,49,50].

In the IHM, both the FH and BH relays participate in stabilizing the folded conformation (Figure 6). The FH relay is located in the BH–FH interface, where R507 of the FH relay forms a network of electrostatic interactions with E370 and Q375 of the BH loop 4 (Figure 6B). The residue at position 507 is Lys in the exon 9a relay and Arg in both exon 9b and 9c relays. Although either Lys or Arg in the exon 9 relay is expected to interact with the BH loop 4, their chemical properties are not the same. Thus, the alternative exon 9 relay in the FH may participate in regulating the stabilization of the shutdown state. In the IHM, the BH relay interacts with segment 2. The aromatic side chain of F517 in the BH relay forms a favorable cation–π interaction with the cationic side chain of R1430 in segment 2 (Figure 6C). F517 is absolutely conserved across all exon 9 variants, suggesting that the cation–π interaction plays a critical and conserved role in the shutdown state of *Drosophila* myosin.

**Exon 11-encoded region**

**Figure 7:**
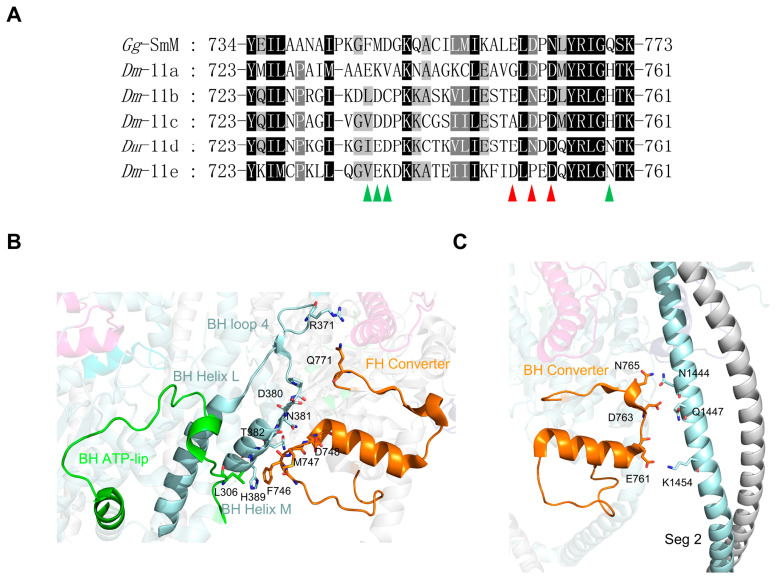
Amino acid sequence and interaction analyses of the exon 11 encoding region. (**A**) Amino acid sequence alignment of chicken smooth muscle myosin and *Drosophila* MHC in the regions encoded by alternative exon 11 of the *Drosophila Mhc* gene. The red arrows indicate the amino acids in BH exon 11 involved in stabilizing the IHM, the green arrows indicate the amino acids in FH exon 11 involved in stabilizing the IHM. (**B**) Interactions between the FH converter, the BH loop 4 and ATP-lip. (**C**) Interactions between the BH converter and segment 2. The color coding is consistent with Figure 3.

The *Drosophila Mhc* gene contains five exon 11 variants (11a, 11b, 11c, 11d and 11e), encoding residues 723 through 761 of Drosophila MHC. The amino acid sequence alignment indicates that this is the least conserved exon, with many different residues among these variants (Figure 7A). Structurally, the exon 11 region is part of the converter, forming a bridge between the relay and the lever arm. It begins near the end of the first of two α-helices in the converter and snakes through a long loop, followed by a long α-helix, and finally ends with a β-sheet adjacent to the end of the lever arm (Figure 7B,C). The converter amplifies the relatively small conformational changes in the motor domain and transmits them to the lever arm. Transgenic *Drosophila*, including chimeric IFM-EC (IFM isoform backbone with the EMB converter domain) and EMB-IC (EMB isoform backbone with the IFM converter domain) both exhibit increased actin–activated ATPase activity [51]. Thus, the alternative exon 11 may modulate the velocity or force of myosin contraction.

In the IHM, both the FH and BH converters participate in stabilizing the folded conformation. The FH converter is located in the FH–BH interface, forming an extensive network of electrostatic interactions and hydrogen bonds with the BH loop 4, helix M, and ATP-lip (Figure 7B). The residues F746-D748 of the FH converter lie near D380 and N381 of the BH loop 4 and T382-H389 of helix M. The FH converter Q771 forms an ionic interaction with R371 of BH loop 4. In addition, the FH converter forms hydrophobic interactions with the BH ATP-lip between L306 and F746. The BH converter residues E761, D763 and N765 form electrostatic interactions with residues N1444, Q1447 and K1454 in segment 2 (Figure 7C). The sequences of the regions encoded by alternative exon 11 are highly variable. Most of the key residues mentioned above, which are critical for IHM formation in the converter, are not conserved in the exon 11 region. We speculated that the alternative exon 11 region variants are likely to affect the stability of the shutdown state of *Drosophila* myosin.

## 4. Discussion

Different myosin-2 paralogs share a common inhibited structure, the IHM, despite their different mechanisms of regulation. The *Mhc* gene in insects produces all muscle MHCs in a way of alternative splicing. We speculated that all the alternative exons might participate in stabilizing the folded conformation. We propose that these alternative exons may be critical to the regulation of insect striated muscle myosin, particularly FH exon 7 and FH exon 9.

The exon 3-encoded portion corresponds to the N-SH3 subdomain in the motor domain, which plays a critical role in transducing chemical energy into movement. While FH exon 3 does not participate in the folded conformation, BH exon 3 contributes significantly to the stability of the IHM, resulting from the electrostatic interactions between BH N-SH3 and segment 2. Exon 7 is located on the surface of the U50 subdomain and forms the ATP-lip, playing a key role in mediating nucleotide access to the active site. BH exon 7 may cooperate with the FH exon 11 region to stabilize the 10S state through a crucial hydrophobic interaction in the BH–FH interface. Exon 9 is the most conserved exon involved in stabilizing the shutdown state. It encodes the entire relay region of MHC, which is critical for myosin energy transduction. Both FH and BH exon 9 regions contribute to stabilizing the folded conformation. FH exon 9 is located in the BH–FH interface and makes electrostatic interactions with BH loop 4. BH exon 9 takes part in the interaction between BH and segment 2, similar to BH exon 3. Interestingly, the interaction between BH exon 9 and segment 2 introduces a novel noncovalent binding force, the cation–π interaction, which contributes to protein stability. Exon 11 is the least conserved exon involved in stabilizing the shutdown state. It encodes a region of the converter. Both FH and BH exon 11 encoding regions participate in the stabilization of the folded conformation too. FH exon 11 is similar to FH exon 9, located in the BH–FH interface, and interacts with the BH ATP-lip, loop 4 and helix M via electrostatic interactions. For BH exon 11, it is blocked by the entanglement of segment 2 with BH. Accordingly, based on the sequence conservation and the fact that the BH exon 7 and FH exon 9 regions are located in the BH–FH interface, the most conserved interaction region responsible for maintaining IHM stability in insect striated muscles, we speculated that BH exon 7 and FH exon 9 are most likely to play the most critical role in the regulation of insect striated muscles.

While this review primarily focuses on the effects of individual binary interactions, it is important to recognize that coordinated changes in multiple exons could lead to synergistic or antagonistic effects on the function and regulation of insect muscle myosin. For instance, the IFI isoform of *Drosophila* muscle myosin exhibits significantly higher motor activity compared to the EMB isoform. However, swapping any one of the four exons that differ between the EMB and IFI isoforms only moderately alters motor activity [21,22,42,43,51], suggesting that the full functional difference between these isoforms arises from the combined effects of all four exon changes. This observation highlights the potential for synergistic interactions between multiple exons, where the collective impact of coordinated changes may be greater than the sum of their individual effects. Conversely, antagonistic interactions could also occur, in which changes in one exon might counteract the effects of changes in another, further complicating the regulation of myosin function. Therefore, it is crucial to consider the combinatorial effects of multiple exon changes, rather than focusing solely on individual binary interactions, when investigating the regulation of insect striated myosin.

Understanding the functional roles of alternative exons in striated muscle myosin requires a combination of in vivo and in vitro approaches. Transgenic *Drosophila* used to be a tractable experimental system for studying how these alternative exons affect the function of striated muscle myosin in vivo. However, the spatial and temporal expression patterns of these alternative exons are complicated in insects, which may limit research on how they regulate striated muscle myosin. Thus, biochemical experiments in vitro using recombinant expressed myosin are necessary to investigate the roles of these alternative exons in regulating striated muscle myosin. Our recent studies show that insect striated muscle myosins can be expressed in Sf9 cells with normal myosin functions, greatly facilitating the study of the structure–function relationship of insect striated muscle myosins [52,53].

Another important issue is to determine the high-resolution structure of insect striated muscle myosin-2 in both its activated and inhibited states. The vast diversity of insects, encompassing over a million described species, presents a unique opportunity to explore the structural intricacies of striated muscle myosin-2 in its inhibited states. This pursuit holds significant promise for advancing our understanding of muscle regulation and contraction mechanisms across a wide range of organisms. We expect that a concerted effort from structural biologists, biochemists and entomologists will elucidate the detailed mechanisms.

## Data Availability

Data sharing not applicable.

## Abbreviations

The following abbreviations are used in this manuscript:

Myosin-2	Class II myosin
MHC	Myosin heavy chain
F-actin	Actin filament
SmM	Smooth muscle myosin
ELC	Essential light chain
RLC	Regulatory light chain
IHM	Interacting heads motif
BH	“Blocked” head
FH	“Free” head
MLCK	Myosin light chain kinase
EMB	Embryonic muscle
IFM	Indirect flight muscle
*Gg*	*Gallus gallus*
*Dm*	*Drosophila melanogaster*
